# Methacrylated gelatin microneedles loaded with cobalt-curcumin nanozymes promote diabetic wound healing by modulating oxidative stress and inflammatory microenvironment

**DOI:** 10.1039/d6ra03654f

**Published:** 2026-08-27

**Authors:** Guixuan You, Wen Chen, Dongbo Xie, Fashen Huang, Yang Shen, Xinke Gu

**Affiliations:** a Yibin Hospital of Integrated Traditional Chinese and Western Medicine Yibin 644100 China; b Orthopedics Department, Yibin Hospital of Integrated Traditional Chinese and Western Medicine Yibin 644100 China; c West China School of Basic Medical Sciences & Forensic Medicine, Sichuan University Chengdu 610041 China nxgxk123@163.com shenyang@scu.edu.cn

## Abstract

Diabetic wounds are characterized by persistent oxidative stress and chronic inflammation, which jointly impair tissue repair. Here, we developed cobalt-curcumin coordination nanoparticles (Co-Cur NPs)-loaded Methacrylated Gelatin (GelMA) microneedles (Co-Cur NPs@MN) for local diabetic wound treatment. Co-Cur NPs were prepared through coordination-driven assembly between cobalt ions and curcumin and encapsulated into photocrosslinked GelMA microneedles. The coordination-derived Co-Cur NPs modulated macrophage-associated inflammatory gene expression. In a streptozotocin (STZ)-induced diabetic rat wound model, Co-Cur NPs@MN accelerated wound closure compared with untreated wounds and topical Co-Cur NPs. Histological analyses showed enhanced granulation tissue formation and collagen deposition. Immunofluorescence further demonstrated a reduced interleukin-1β (IL-1β)-positive area and an increased interleukin-10 (IL-10)-positive area in treated wounds. These findings suggest that Co-Cur NPs@MN provides an effective local therapeutic strategy for diabetic wound repair by integrating nanozyme-mediated oxidative stress regulation with inflammatory microenvironment modulation.

## Abstract 2

Co-Cur NPs@MN for promoting diabetic wound healing.

## Graphical abstract

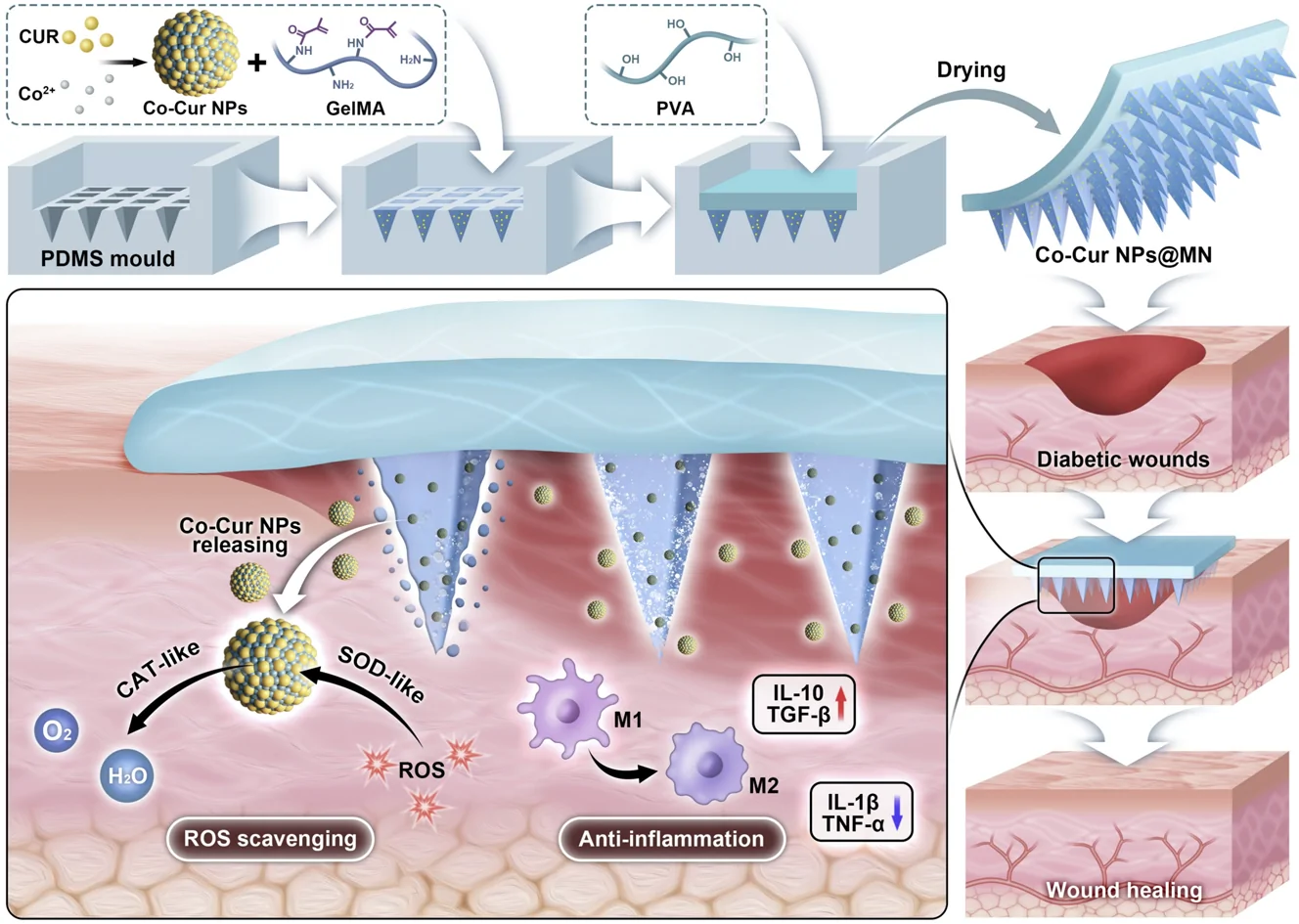

## 1. Introduction

Chronic wounds constitute a significant global public health burden, with escalating incidence rates and growing recognition of their substantial socioeconomic impact emerging as an increasingly critical clinical challenge in recent years.^1^ Among these, diabetic foot ulcers have garnered particular attention due to their high prevalence, refractory healing characteristics, and substantial amputation risk. The unique pathophysiology of the diabetic wound microenvironment is characterized by persistent hyperglycemia-induced excessive oxidative stress, sustained chronic inflammatory responses, and impaired angiogenesis, which collectively establish a vicious cycle that severely compromises normal wound healing.^2,3^ Despite the implementation of various therapeutic modalities in clinical practice, including surgical debridement, negative pressure wound therapy, hyperbaric oxygen therapy, and diverse wound dressings, current treatment strategies frequently fail to achieve satisfactory outcomes for complex diabetic wounds.^4–6^ Consequently, developing innovative therapeutic strategies capable of simultaneously modulating oxidative stress and the inflammatory microenvironment holds significant clinical importance for disrupting this pathological cycle and promoting diabetic wound healing.

Nanozymes, a class of nanomaterials possessing enzyme-like catalytic activities, have garnered extensive attention in the biomedical field in recent years. Compared to natural enzymes, nanozymes offer advantages including superior stability, facile synthesis, cost-effectiveness, and amenability to functional modification.^7^ Notably, nanozymes exhibiting superoxide dismutase (SOD)-like and catalase (CAT)-like activities can efficiently scavenge reactive oxygen species (ROS), demonstrating tremendous potential in anti-inflammatory applications and tissue repair promotion.^8^ Curcumin, a natural polyphenolic compound, exhibits favorable anti-inflammatory, antioxidant, and immunomodulatory properties.^9^ However, its poor aqueous solubility and low bioavailability severely constrain its clinical application. The construction of metal–curcumin coordination nanozymes not only significantly improves curcumin solubility and stability but also confers unique enzyme-like catalytic activities through coordination between metal ions and curcumin phenolic hydroxyl groups.^10–12^ Among various metal ions, cobalt ions have attracted particular interest due to their distinctive properties. First, cobalt is a biologically relevant metal element involved in vitamin B12 metabolism. However, the biological effects and safety profiles of cobalt-containing materials are highly dependent on their chemical forms, doses, and exposure conditions.^13^ Second, cobalt ions possess variable valence state, and this redox property endows cobalt-based nanomaterials with excellent enzyme-like catalytic activities,^14^ enabling potential SOD-like and CAT-like catalytic activities involved in ROS regulation. Furthermore, cobalt ions exhibit certain antibacterial activities, capable of exerting bactericidal effects through disruption of bacterial cell membrane integrity and interference with bacterial metabolism.^15^ In this design, curcumin serves as an organic ligand with β-diketone/enol and phenolic hydroxyl groups, whereas cobalt ions provide coordination nodes to form nanoscale cobalt-curcumin assemblies. Therefore, Co-Cur NPs are expected to combine the free-radical neutralization capability of curcumin with the redox-modulating potential of cobalt coordination, thereby providing a promising material basis for regulating oxidative stress and inflammatory microenvironments in diabetic wounds.

Nevertheless, the application of nanomedicines in wound therapy continues to face challenges including low delivery efficiency and short retention times. Microneedles, as an emerging transdermal drug delivery system, possess characteristics of minimal invasiveness, painless administration, and controlled release, enabling precision drug delivery across the skin barrier.^16^ GelMA, a photo-crosslinkable hydrogel, exhibits favorable biocompatibility, biodegradability, and cell responsiveness, finding extensive applications in tissue engineering and drug delivery.^17^ GelMA-based microneedle systems not only provide stable carriers for nanozymes but also achieve controlled sustained release through photo-crosslinking, thereby extending drug action duration at wound sites. Additionally, polyvinyl alcohol (PVA) has also been widely used in biomedical polymer blends and membrane systems because of its film-forming ability, flexibility, and processability. These properties support its use as a backing layer to improve the structural integrity and handling convenience of microneedle patches.^18–21^

**Scheme 1:**
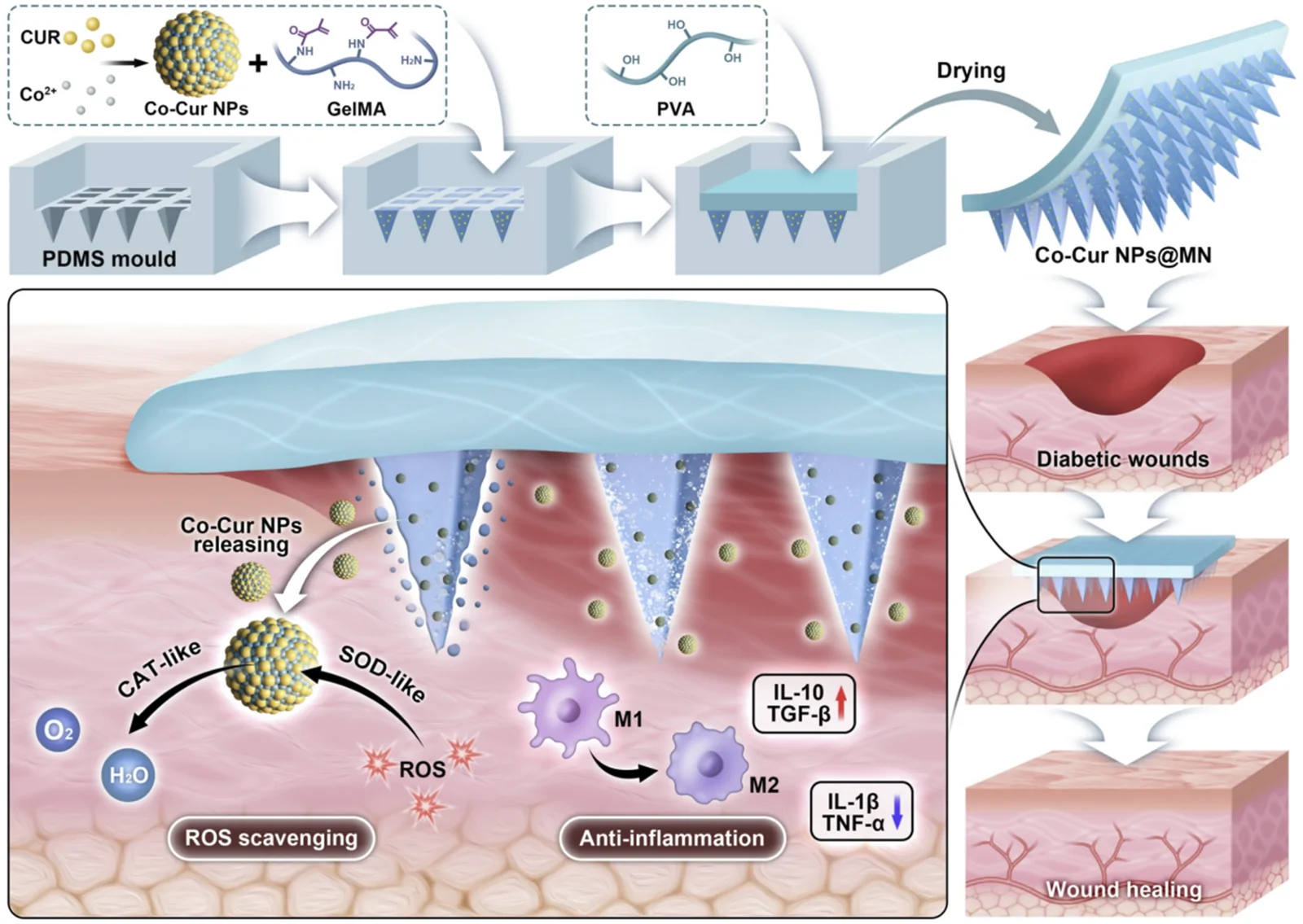
Schematic illustration of Co-Cur NPs@MN for promoting diabetic wound healing.

Based on the aforementioned background, this study proposes an integrated therapeutic strategy by constructing a Co-Cur NPs@MN system for diabetic wound treatment (Scheme 1). This system combines the ROS-regulating nanozyme-like activity and inflammation-modulating capability of Co-Cur NPs with the localized administration advantages of GelMA microneedles. Co-Cur NPs were designed as a metal–polyphenol coordination nanozyme to alleviate oxidative stress and regulate the inflammatory microenvironment of diabetic wounds. Meanwhile, GelMA microneedles provide a minimally invasive and localized delivery approach to facilitate the retention and therapeutic action of Co-Cur NPs at wound sites. The therapeutic efficacy of Co-Cur NPs@MN was systematically evaluated through *in vitro* cellular experiments and an *in vivo* diabetic rat wound model, providing insights into the potential of coordination nanozyme-based microneedle systems for diabetic wound repair.

## 2. Materials and methods

### 2.1 Reagents and materials

Polyvinylpyrrolidone (PVP) and STZ were purchased from Macklin (Shanghai, China). Cobalt chloride hexahydrate (CoCl_2_·6H_2_O) was obtained from Chengdu Huachun Technology Co., Ltd (Chengdu, China). Curcumin powder (purity: 98.63%) was purchased from MedChemExpress (New Jersey, USA). Phorbol 12-myristate 13-acetate (PMA) and lipopolysaccharide (LPS) were purchased from Sigma-Aldrich (St. Louis, MO, USA). Interferon-γ (IFN-γ) was obtained from PeproTech (New Jersey, USA). Calcein AM/PI cell viability staining kit, hematoxylin and eosin (H&E) staining kit, and Masson staining kit were purchased from Beyotime Biotechnology (Shanghai, China). GelMA and PVA were purchased from Suzhou Yongqinquan Intelligent Equipment Co., Ltd (EFL, Suzhou, China). IL-10 and IL-1β primary antibody was purchased from Servicebio (Wuhan, China). CD86 and CD206 primary antibody was purchased from Abcam (Cambridge, UK). Fluorescent-labeled secondary antibodies were obtained from Jackson ImmunoResearch (West Grove, PA, USA). Tyramide signal amplification reagents were purchased from Runnerbio (Shanghai, China). Reverse transcription quantitative polymerase chain reaction (RT-qPCR) primers were synthesized by Accurate Biology (Hunan, China). DPPH and ABTS free radical scavenging kits were purchased from Beyotime Biotechnology (Shanghai, China).

### 2.2 Preparation of Co-Cur NPs

Cobalt-curcumin nanozymes (Co-Cur NPs) were prepared *via* a coordination reaction according to previously reported metal–polyphenol/metal–curcumin coordination strategies with minor modifications.^10,22,23^ Briefly, 132 mg PVP was dissolved in 10 mL methanol under magnetic stirring until completely transparent. Subsequently, 20 mg cobalt chloride hexahydrate (CoCl_2_·6H_2_O, 0.0840 mmol) was dissolved in 1 mL methanol and stirred until complete dissolution. Separately, 20 mg curcumin (0.0543 mmol) was dissolved in 2 mL methanol and stirred until complete dissolution. Based on these feeding amounts, the molar ratio was approximately 1.55 : 1. The CoCl_2_ solution was slowly added dropwise to the PVP-methanol solution under continuous stirring. Subsequently, the curcumin solution was slowly added dropwise to the above mixture. The mixture was continuously stirred at room temperature for 3 h to ensure sufficient coordination reaction. Finally, the mixture was dialyzed against deionized water overnight to remove unreacted small molecules, yielding the Co-Cur NPs dispersion. The obtained Co-Cur NPs dispersion was transferred into sterile polypropylene centrifuge tubes, sealed, protected from light, and stored at 4 °C until further use. Before use, the dispersion was gently vortexed to ensure uniform resuspension.

### 2.3 Characterization of Co-Cur NPs

The physicochemical properties of Co-Cur NPs were characterized by dynamic light scattering (DLS), zeta potential analysis, transmission electron microscopy (TEM)/high-resolution TEM (HRTEM), high-angle annular dark-field scanning TEM (HAADF-STEM), energy-dispersive spectroscopy (EDS) elemental mapping, and Fourier transform infrared spectroscopy (FTIR) according to commonly used characterization strategies for coordination nanoparticles and nanozymes.^22–24^ Particle size distribution and zeta potential of Co-Cur NPs were measured using a Malvern Zetasizer Nano ZS-90 (Malvern Instruments, UK). To evaluate the colloidal stability in physiological environments, Co-Cur NPs were incubated in PBS or PBS containing 10% FBS for 7 days. The hydrodynamic size changes were monitored at days 0, 1, 3, and 7 using dynamic light scattering (DLS). Samples were sonicated for 3 minutes before measurement, with scattered light detection at 90° and test temperature at 25 °C. Each sample was measured in triplicate, and average values were reported. TEM analysis: Co-Cur NPs were dispersed in ethanol solution and sonicated, followed by dropping several drops of dispersion onto copper grids and air-drying before observation. Nanoparticle morphology and size were observed using a transmission electron microscope at 200 kV acceleration voltage. HRTEM and HAADF-STEM observations were also performed, along with EDS elemental distribution analysis. FTIR analysis: tests were performed using an FTIR spectrometer equipped with an attenuated total reflectance (ATR) accessory. Air background was first scanned, then samples were placed against the ATR crystal surface for infrared spectrum acquisition. Resolution was 4 cm^−1^, scan number was 32, and test wavenumber range was 400–4000 cm^−1^. The elemental composition and valence distribution were determined *via* X-ray photoelectron spectroscopy (XPS).

### 2.4 Free radical scavenging capacity assay of Co-Cur NPs

The antioxidant capacity of Co-Cur NPs was evaluated using DPPH and ABTS free radical scavenging assays.^25,26^ Different concentrations (1, 2.5, 5, 10, 20 µg mL^−1^) of Co-Cur NPs were added to solutions containing DPPH or ABTS, respectively. After incubation at room temperature for 30 minutes, absorbance changes before and after reaction were measured using an ultraviolet-visible (UV-visible) spectrophotometer. Free radical scavenging rates were calculated according to the following formula: scavenging rate (%) = (*A*_0_ − *A*_1_)/*A*_0_ × 100%, where *A*_0_ represents control group absorbance and *A*_1_ represents sample group absorbance. The SOD-like and CAT-like activities of Co-Cur NPs (6.25–200 µg mL^−1^) were measured using commercial assay kits according to the manufacturers' instructions (Beyotime, China).

### 2.5 Preparation of Co-Cur NPs@MN

Co-Cur NPs@MN patches were fabricated using a mold-casting and photocrosslinking strategy adapted from previously reported GelMA-based and hydrogel microneedle systems.^17,27,28^ Co-Cur NPs solution was uniformly mixed with 10% (w/v) GelMA hydrogel precursor solution containing 0.05% LAP photoinitiator, then the mixture was dropped onto polydimethylsiloxane (PDMS) microneedle molds and subjected to 3–4 vacuum extraction cycles at room temperature to remove bubbles, ensuring complete solution filling of needle tips. Subsequently, molds were placed in a 30–35 °C oven for multiple heating and concentration cycles. After UV 405 nm photo-curing, 20% (w/v) PVA solution was dropped onto the microneedle base to form the backing layer. PVA was selected because of its film-forming ability, flexibility, and broad use in biomedical polymeric blends and membrane systems.^18–21^ Finally, after drying at 30–35 °C for 12 hours, demolding was performed to obtain Co-Cur NPs@MN. After demolding, the dried Co-Cur NPs@MN patches were individually placed in sterile light-protected containers with desiccant, sealed, and stored at 4 °C under dry conditions until use. Before application, the microneedle patches were equilibrated to room temperature.

### 2.6 Characterization of Co-Cur NPs@MN

Scanning electron microscopy (SEM) observation was performed to evaluate the overall array morphology and needle-tip integrity according to previously reported microneedle characterization methods.^17,27,28^ The morphology of Co-Cur NPs@MN was observed using a field-emission scanning electron microscope (FEI Nova NanoSEM). Samples were sputter-coated with gold, and microneedle array overall morphology and needle tip structure were observed at 5 kV acceleration voltage. The mechanical performance of Co-Cur NPs@MN was evaluated using the TA. XT Plus texture analyzer (Stable Micro Systems, UK). The microneedle patch consisted of a 15 × 15 array with 225 microneedles. Each microneedle had a height of 800 µm, an inter-needle spacing of 720 µm, and a square base of 360 µm × 360 µm. During testing, the whole microneedle patch was placed on the platform with the needle tips facing upward, and a flat compression probe was moved downward at a constant speed of 2 mm min^−1^. The compression force was recorded as a function of displacement. The mechanical strength was evaluated at a displacement of 500 µm, and the force per needle was calculated by dividing the total compression force by the number of microneedles. For the skin-insertion test, Co-Cur NPs@MN patches were applied to ex vivo pigskin for 10 seconds under, and the resulting microchannels were imaged after patch removal.

### 2.7 Cell culture

Human umbilical vein endothelial cells (HUVECs) were cultured in Dulbecco's Modified Eagle Medium (DMEM) containing 10% fetal bovine serum (FBS), 100 U mL^−1^ penicillin, and 100 µg mL^−1^ streptomycin. Human monocytic leukemia cells (THP-1) were cultured in Roswell Park Memorial Institute (RPMI) 1640 medium containing 10% FBS. All cells were maintained in a humidified incubator at 37 °C with 5% CO_2_.

### 2.8 Establishment of macrophage polarization model

THP-1 macrophage differentiation and inflammatory polarization were performed based on a previously reported THP-1-derived macrophage polarization protocol.^29^ THP-1 cells were first seeded at a density of 5 × 10^5^ cells per mL in medium containing PMA (100 ng mL^−1^) and treated for 48 hours to induce macrophage differentiation and adherent growth. Subsequently, the medium was replaced with RPMI 1640 containing 10% FBS, supplemented with 1000 ng mL^−1^ LPS and 50 ng mL^−1^ IFN-γ, and induced for 24 hours to establish the M1-type inflammatory macrophage model. Cells were then treated with medium (control group), curcumin (Cur group), or Co-Cur NPs for 24 hours before collection for subsequent analyses.

### 2.9 RT-qPCR analysis

For *in vitro* analysis, total RNA was extracted from treated THP-1-derived macrophages using the TRIzol method. For *in vivo* analysis, wound tissues collected on day 14 were homogenized, and total RNA was extracted using the TRIzol method. RNA was reverse-transcribed to complementary DNA (cDNA) using a reverse transcription kit. The messenger RNA (mRNA) expression levels of inflammation-related cytokines and macrophage-associated markers were detected using a real-time fluorescence quantitative PCR system (Bio-Rad). Glyceraldehyde-3-phosphate dehydrogenase (GAPDH) served as the internal reference gene, and relative expression levels of target genes were calculated using the 2^−ΔΔCt^ method.^30^

### 2.10 Live-dead cell staining

HUVECs were seeded at appropriate density in 24-well plates and treated with medium (control group), Co-Cur NPs, or Co-Cur NPs@MN extract for 24 hours. Staining was performed according to the calcein AM/PI cell viability staining kit instructions: culture medium was removed, cells were washed with phosphate-buffered saline (PBS), and staining working solution containing calcein AM and PI was added and incubated at 37 °C in the dark for 30 minutes. Observations and photography were performed using an inverted fluorescence microscope, with viable cells exhibiting green fluorescence and dead cells exhibiting red fluorescence.

### 2.11 Establishment of diabetic rat model

The STZ-induced diabetic rat model was established according to a previously reported diabetic wound-healing model.^31^ Male Sprague-Dawley rats (250–300 g) were used and acclimated for one week before establishing the diabetic model. After 12 hours of fasting, rats received intraperitoneal injection of 1% STZ solution (60 mg kg^−1^, dissolved in 0.1 M citric acid-sodium citrate buffer, pH 4.5). After 7 days of continued feeding, fasting blood glucose was measured from tail vein blood. Rats with sustained fasting blood glucose levels ≥16.7 mmol L^−1^ were considered successfully established diabetic models.

### 2.12 Establishment of diabetic wound model and treatment

The full-thickness diabetic wound model was established based on a previously reported STZ-induced diabetic rat wound model.^31^ Diabetic rats were anesthetized *via* isoflurane inhalation, and dorsal hair was removed and disinfected. A 10 mm diameter circular punch was used to create two full-thickness skin defect wounds on the rat dorsum. Diabetic rats were randomly allocated to five groups (*n* = 3 animals per group): control, free curcumin (Cur), Co-Cur NPs, blank GelMA microneedles (MN), and Co-Cur NPs@MN. The doses of Cur and Co-Cur NPs were matched. Two wounds were created in each animal and received the same assigned treatment. Measurements from the two wounds were averaged to obtain one value per animal. Wound-area measurement and subsequent histological analyses were performed by investigators blinded to group allocation. All wounds were covered with 3 M Tegaderm transparent dressing for protection. Wound healing was documented photographically at days 0, 3, 7, 10, and 14 post-surgery, and wound areas were measured using ImageJ software to calculate the relative wound area. Relative wound area (%) = *A_t_*/*A*_0_ × 100%, where *A*_0_ represents the initial wound area and *A_t_* represents the wound area at each time point.

### 2.13 Histological analysis

H&E and Masson staining were performed to evaluate re-epithelialization, granulation tissue formation, and collagen deposition, as described in previous wound-healing studies.^32,33^ Upon treatment completion (day 14), rats were euthanized and wound tissues were collected. Tissue samples were fixed in 4% paraformaldehyde for 48 hours, dehydrated through graded ethanol, cleared in xylene, and embedded in paraffin. Paraffin blocks were sectioned into 7 µm thick serial sections. H&E staining: sections were stained according to the H&E staining kit instructions to observe overall wound tissue morphology, degree of re-epithelialization, and granulation tissue formation. Granulation tissue thickness was measured using ImageJ software. Masson trichrome staining: sections were stained according to the Masson staining kit instructions to observe collagen deposition (collagen fibers appear blue). Collagen deposition percentage was analyzed using ImageJ software.

### 2.14 Immunofluorescence staining

IL-1β, IL-10, CD86 and CD206 were selected as representative inflammatory and anti-inflammatory cytokines associated with the inflammatory wound microenvironment and macrophage-related immune regulation.^34–36^ Paraffin sections were deparaffinized and rehydrated, then placed in antigen retrieval solution and pressure-cooked for 3 minutes. Endogenous peroxidase was blocked with 3% H_2_O_2_ for 10 minutes, followed by three PBS washes (5 minutes each). 5% bovine serum albumin (BSA) blocking solution was added and incubated at room temperature for 20 minutes. Diluted IL-1β primary antibody (1 : 2500), IL-10 primary antibody (1 : 800), CD86 primary antibody (1 : 500) and CD206 primary antibody (1 : 5000) was applied and incubated overnight at 4 °C. After PBS washing, fluorescent-labeled secondary antibody (1 : 400) was added and incubated at 37 °C for 60 minutes. Following PBS washing, tyramide signal amplification reagent was added and incubated at room temperature for 20 minutes. Finally, 4′,6-diamidino-2-phenylindole (DAPI) was added for nuclear counterstaining and incubated at room temperature in the dark for 10 minutes before mounting. Observations and photography were performed using a fluorescence microscope, and positive area percentages were analyzed using ImageJ software.

### 2.15 Biosafety evaluation

Upon treatment completion, blood samples were collected from the orbital venous plexus of rats in the control and Co-Cur NPs@MN groups for complete blood count (CBC) and blood biochemistry analyses. CBC parameters included white blood cell count (WBC), red blood cell count (RBC), and platelet count (PLT). Blood biochemistry parameters included liver function indices, including alanine aminotransferase (ALT) and aspartate aminotransferase (AST), and renal function indices, including uric acid (UA), creatinine (CREA), urea (UREA), and blood urea nitrogen (BUN).

### 2.16 Statistical analysis

All experimental data are presented as mean ± standard deviation (SD). For the *in vivo* experiments, the animal was considered the experimental unit (*n* = 3 animals per group). When two wounds from the same animal contributed to the same endpoint, their measurements were averaged before statistical analysis. Longitudinal relative wound area trajectories were presented descriptively without inferential statistical analysis. Comparisons among treatment groups at predefined single-time-point endpoints were performed using one-way ANOVA followed by Tukey's post hoc multiple-comparisons test. Comparisons between two groups were performed using an independent-samples *t*-test. Statistical analyses were conducted using GraphPad Prism 9.0.

## 3. Results and discussion

### 3.1 Synthesis and characterization of Co-Cur NPs

**Fig. 1:**
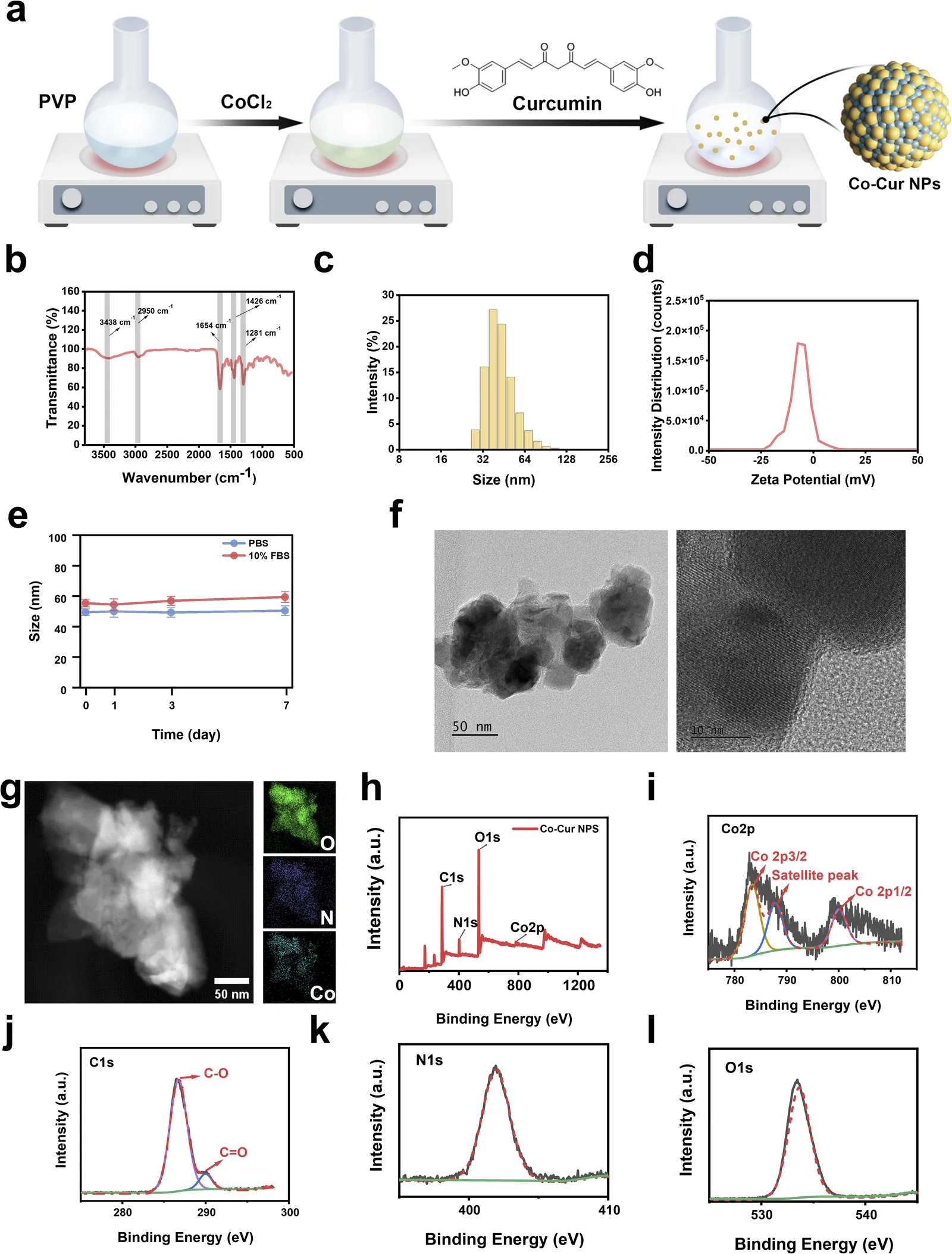
Synthesis and characterization of Co-Cur NPs. (a) Schematic illustration of the preparation of Co-Cur NPs. (b) FTIR spectrum. (c) Hydrodynamic size distribution. (d) Zeta-potential distribution. (e) Time-dependent hydrodynamic diameter of Co-Cur NPs during incubation in PBS and 10% FBS for 7 days. (f) TEM and HRTEM images of Co-Cur NPs. Scale bars: 50 nm and 10 nm. (g) HAADF-STEM image and corresponding elemental maps of O, N, and Co. Scale bar: 50 nm. (h) XPS survey spectrum. (i–l) High-resolution XPS spectra of Co 2p (i), C 1s (j), N 1s (k), and O 1s (l).

This study employed a coordination reaction strategy to prepare Co-Cur NPs. As illustrated in Fig. 1a, PVP was dissolved in methanol as a stabilizer, followed by sequential addition of cobalt chloride (CoCl_2_) and curcumin, forming Co-Cur NPs through coordination between Co^2+^ and the phenolic hydroxyl/β-diketone groups of curcumin.^22^ FTIR analysis (Fig. 1b) revealed O–H stretching vibration peaks near 3438 cm^−1^, C=C and C=O characteristic absorption peaks in the 1700–1400 cm^−1^ region, and C–O stretching vibration peaks near 1281 cm^−1^. These characteristic peaks align with the structural features of curcumin, confirming its incorporation into the nanozyme system, while partial peak shifts suggest metal–ligand coordination bond formation. DLS results (Fig. 1c) demonstrated that Co-Cur NPs exhibited an average particle size of approximately 45.2 nm with a relatively uniform size distribution (PDI = 0.195). Zeta-potential analysis revealed a surface potential of approximately −6.9 mV (Fig. 1d). Because this relatively weak surface charge alone was insufficient to demonstrate colloidal stability, time-dependent DLS measurements were performed. The hydrodynamic diameter remained relatively stable during 7 days of incubation in PBS and FBS (Fig. 1e), supporting short-term colloidal stability under the tested conditions. TEM and HRTEM images (Fig. 1f) revealed the nanoparticle morphology and distinct lattice fringes.^24^ The HAADF-STEM image and corresponding elemental maps (Fig. 1g) demonstrated the co-distribution of O, N, and Co within the nanoparticles. XPS analysis further confirmed the presence of C, N, O, and Co (Fig. 1h). The high-resolution Co 2p spectrum showed Co 2p_3/2_ and Co 2p_1/2_ signals together with characteristic satellite features (Fig. 1i), while the C 1s, N 1s, and O 1s spectra further supported the presence of PVP and oxygen-containing organic ligands (Fig. 1j–l). The FTIR, elemental-mapping, and XPS results collectively support the construction of the Co-Cur coordination structure. Collectively, these results confirm the preparation of structurally stable and uniformly sized Co-Cur NPs, providing a foundation for subsequent biological evaluation.

### 3.2 Free radical scavenging capacity of Co-Cur NPs and fabrication and characterization of Co-Cur NPs@MN

**Fig. 2:**
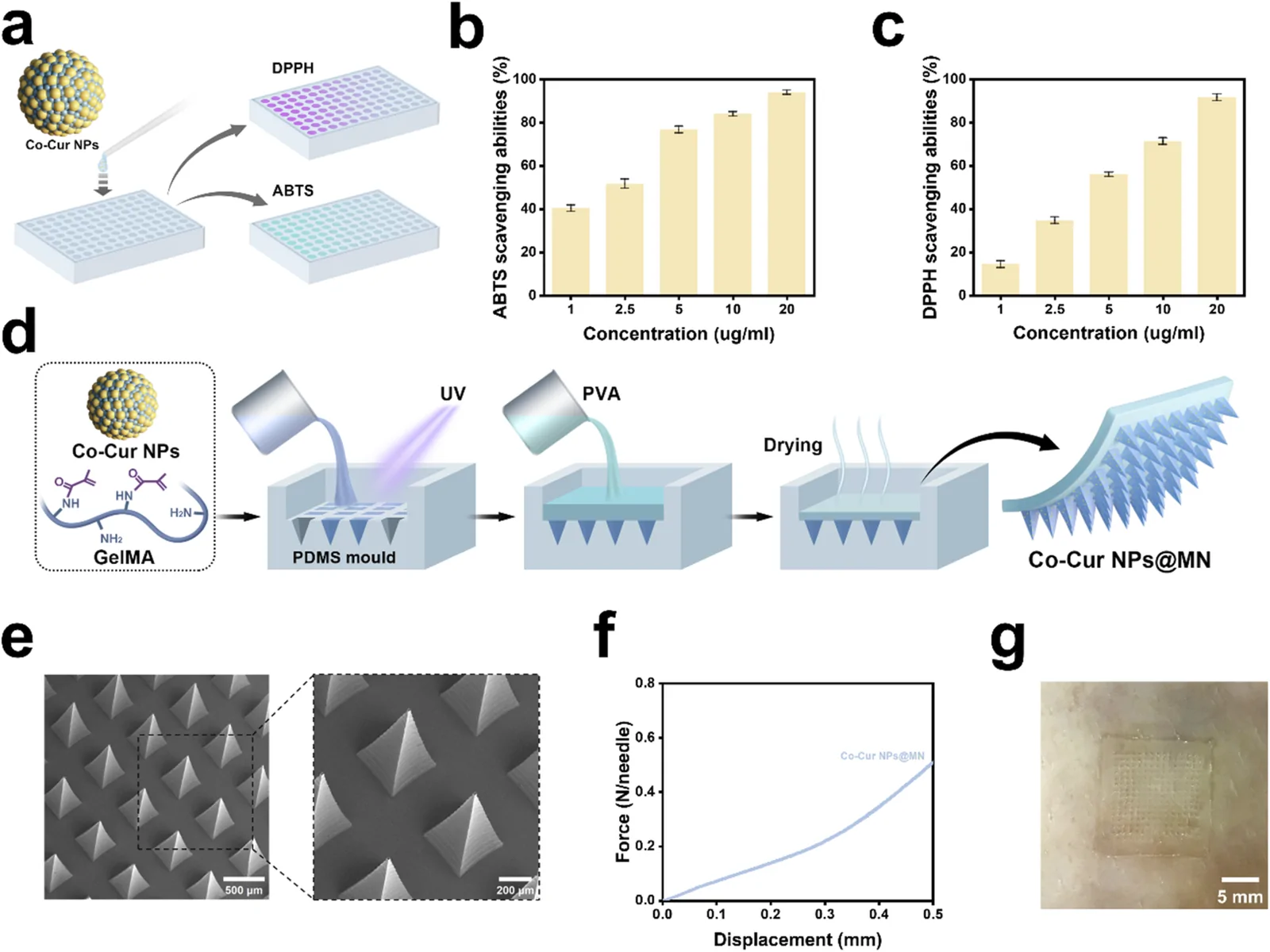
Radical-scavenging activity and characterization of Co-Cur NPs@MN. (a) Schematic illustration of the DPPH and ABTS assays. (b and c) ABTS (b) and DPPH (c) radical-scavenging activities of Co-Cur NPs at the indicated concentrations. (d) Schematic illustration of Co-Cur NPs@MN fabrication. (e) SEM images of the microneedle array and needle tips. Scale bars: 500 and 200 µm. (f) Force-displacement curve normalized to the number of microneedles. (g) Representative image of the microchannel array formed in ex vivo skin after microneedle application scale bar: 5 mm.

To evaluate the antioxidant properties of Co-Cur NPs, DPPH and ABTS free radical scavenging assays were performed (Fig. 2a). As shown in Fig. 2b, Co-Cur NPs exhibited concentration-dependent ABTS free radical scavenging capacity, with scavenging rates increasing from approximately 40% to 95% as concentrations increased from 1 µg mL^−1^ to 20 µg mL^−1^. Similarly, Co-Cur NPs demonstrated significant DPPH free radical scavenging effects (Fig. 2c), with scavenging rates of approximately 15% at 1 µg mL^−1^ and reaching approximately 90% at 20 µg mL^−1^. This radical-scavenging behavior may be related to the combined contribution of curcumin and cobalt-curcumin coordination. The phenolic hydroxyl groups of curcumin can participate in free-radical neutralization through hydrogen donation, while coordination with cobalt ions may alter the local electronic environment of curcumin and improve its dispersion in aqueous systems.^10,22,23,25^ DPPH and ABTS assays are commonly used as preliminary chemical assays for evaluating the radical-scavenging capacity of antioxidant nanomaterials.^26^ To further evaluate the enzyme-mimetic activity of Co-Cur NPs, CAT-like and SOD-like activity assays were performed. Both activities increased with increasing Co-Cur NP concentration from 6.25 to 200 µg mL^−1^ (SI Fig. S1), providing additional evidence of concentration-dependent enzyme-mimetic activity under the applied assay conditions. Therefore, these results indicate that Co-Cur NPs possess concentration-dependent chemical radical-scavenging ability, consistent with the design rationale derived from metal–curcumin nanozyme systems.

Subsequently, Co-Cur NPs were loaded into GelMA microneedles to fabricate Co-Cur NPs@MN (Fig. 2d). Co-Cur NPs were mixed with GelMA hydrogel precursor solution, injected into microneedle molds, and photo-cured under UV 405 nm illumination, followed by addition of 20% PVA solution to form a backing layer and drying for demolding to obtain Co-Cur NPs@MN. SEM images (Fig. 2e) revealed well-organized microneedle arrays with pyramidal needle bodies and intact sharp tips, indicating that the mold-casting and photocrosslinking process successfully preserved the microneedle architecture. To further evaluate the mechanical performance of the microneedle patch, compression testing was performed using a texture analyzer. As shown in Fig. 2f, the force increased progressively with increasing displacement and reached approximately 0.51 N/needle at a displacement of 500 µm, indicating that Co-Cur NPs@MN possessed sufficient mechanical integrity for handling and application. As a photocrosslinked hydrogel, GelMA forms a three-dimensional network capable of encapsulating Co-Cur NPs, while the PVA backing layer was introduced to improve patch integrity and handling convenience during demolding and application.^37^ To further evaluate the delivery performance of Co-Cur NPs@MN, a skin-insertion experiment was performed. After microneedle application, a regular array of visible microchannels was observed on the skin surface (Fig. 2g), supporting the skin-insertion ability of the microneedles.

### 3.3 Effects of Co-Cur NPs on macrophage inflammation-related gene expression

**Fig. 3:**
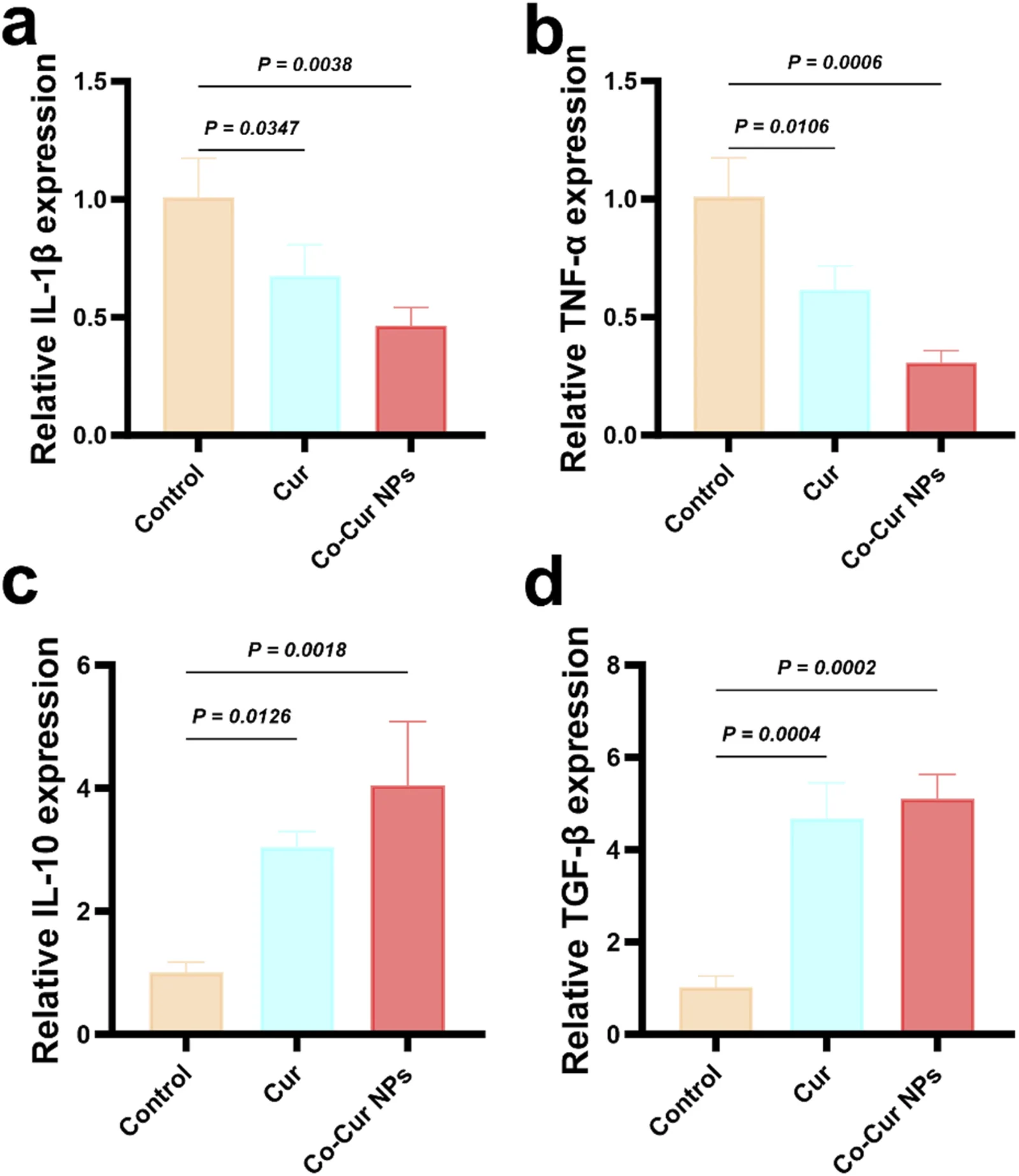
Effects of Co-Cur NPs on inflammation-related gene expression in macrophages. (a and b) Relative mRNA expression levels of pro-inflammatory cytokines IL-1β (a) and TNF-α (b). (c and d) Relative mRNA expression levels of anti-inflammatory cytokines IL-10 (c) and TGF-β (d).

Macrophages play a pivotal role in wound healing, with phenotypes categorized into pro-inflammatory M1 and reparative M2 subtypes. In diabetic wounds, macrophages frequently remain arrested in the M1 phenotype, continuously releasing pro-inflammatory cytokines such as IL-1β and TNF-α, leading to chronic inflammatory states that severely impede wound healing.^38^ To evaluate the anti-inflammatory effects of Co-Cur NPs, RT-qPCR was employed to assess their influence on inflammatory cytokine expression in inflammatory macrophages (Fig. 3). Compared to the control group, IL-1β mRNA expression levels were significantly downregulated in both the Cur group and Co-Cur NPs group, with the Co-Cur NPs group demonstrating more pronounced downregulation (Fig. 3a). Similarly, TNF-α expression exhibited comparable trends (Fig. 3b), with both Cur and Co-Cur NPs groups significantly lower than the control group. Regarding anti-inflammatory factors, IL-10 expression was significantly upregulated in both Cur and Co-Cur NPs groups (Fig. 3c), reaching approximately 3-fold and 4-fold that of the control group, respectively. Transforming growth factor-β (TGF-β) expression displayed similar upregulation trends (Fig. 3d).

These results indicate that Co-Cur NPs modulate macrophage inflammation-related cytokine expression profiles by downregulating pro-inflammatory factors (IL-1β, TNF-α) and upregulating anti-inflammatory factors (IL-10, TGF-β). Notably, the anti-inflammatory effects of the Co-Cur NPs group surpassed those of curcumin alone, potentially attributable to several factors. First, coordination structure formation between cobalt ions and curcumin enhanced curcumin stability and bioavailability. Second, the intrinsic enzyme-like catalytic activity of cobalt ions enables scavenging ROS. Additionally, the nano-scale dimensions of Co-Cur NPs favor cellular uptake, increasing intracellular drug concentration. The significant upregulation of IL-10 and TGF-β suggests that Co-Cur NPs can promote macrophage polarization toward the M2 phenotype, bearing significant implications for inflammation resolution and tissue repair in diabetic wounds.

### 3.4 Cytocompatibility evaluation of Co-Cur NPs and Co-Cur NPs@MN

**Fig. 4:**
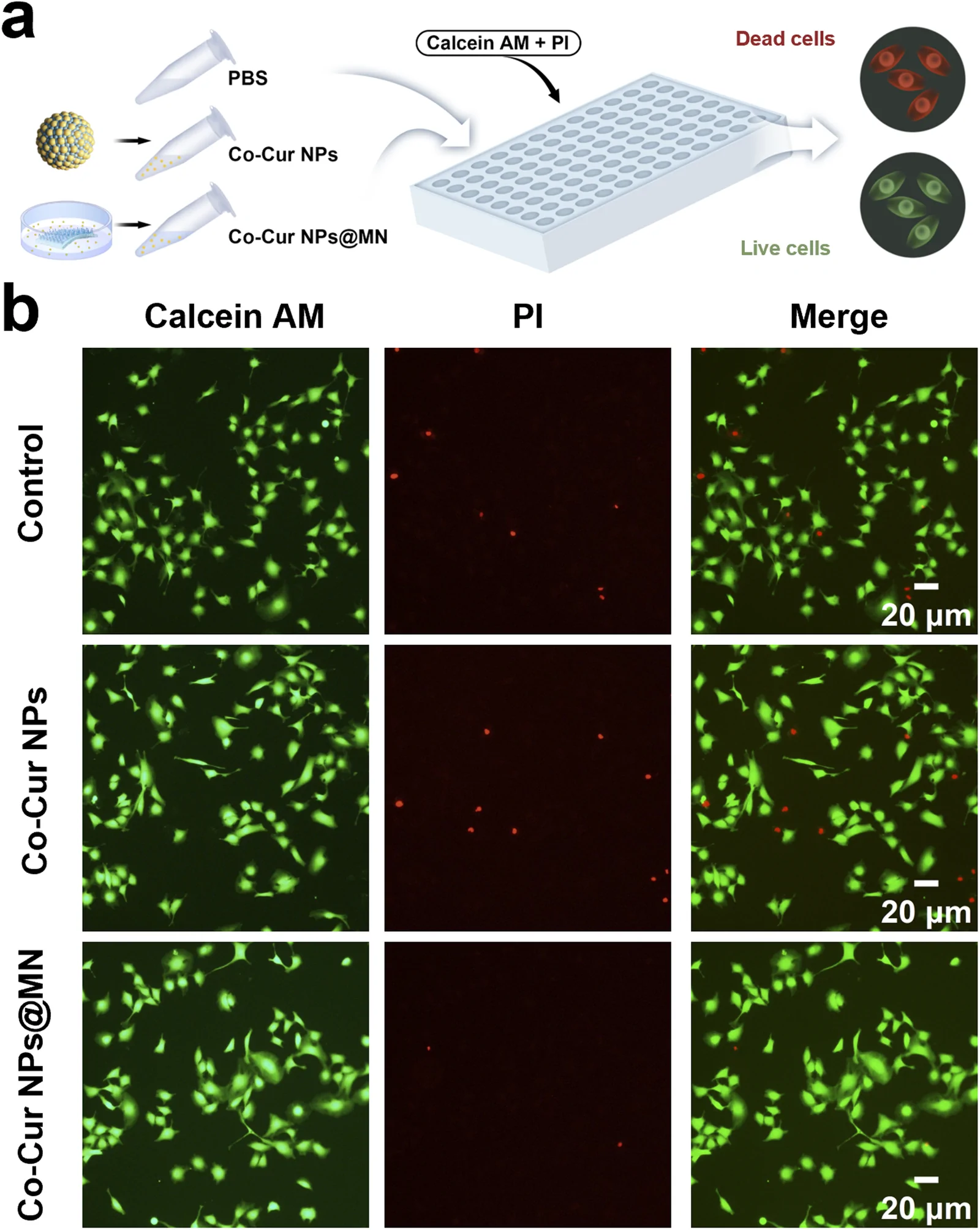
Cytocompatibility evaluation of Co-Cur NPs and Co-Cur NPs@MN. (a) Schematic illustration of calcein AM/PI live/dead cell staining assay. (b) Fluorescence images of live/dead staining of HUVECs in different treatment groups (control, Co-Cur NPs, Co-Cur NPs@MN). Green: live cells; red: dead cells scale bar: 20 µm.

Cytocompatibility represents a critical indicator for evaluating biomaterial safety. HUVECs, as vascular endothelial cells, play essential roles during the neovascularization phase of wound healing, making them representative cell models. This study employed calcein AM/PI live-dead cell staining to assess the cytotoxicity of Co-Cur NPs and Co-Cur NPs@MN toward HUVECs (Fig. 4a). As shown in Fig. 4b, HUVECs in the control, Co-Cur NPs, and Co-Cur NPs@MN groups all exhibited abundant green fluorescence (viable cells) with minimal red fluorescence (dead cells). No significant differences in viable cell proportions were observed among the three groups, and cells maintained normal morphology with characteristic spindle-shaped or polygonal endothelial cell appearance. Calcein AM/PI dual staining results demonstrated that cell viability following Co-Cur NPs and Co-Cur NPs@MN treatment was comparable to the control group, indicating that both materials possess favorable cytocompatibility without inducing significant cytotoxicity. This favorable biocompatibility may be attributed to several factors: first, curcumin, as a natural plant extract, inherently exhibits low cytotoxicity; second, cobalt, as an essential trace element in the human body, does not cause cellular damage at appropriate concentrations; additionally, GelMA hydrogel, derived from gelatin, possesses excellent biocompatibility and cell affinity. Notably, cell viability in the Co-Cur NPs@MN group was comparable to the Co-Cur NPs group, indicating that the microneedle fabrication process did not adversely affect nanozyme biocompatibility. These results provide safety assurance for subsequent *in vivo* applications of Co-Cur NPs@MN.

### 3.5 Wound healing analysis

**Fig. 5:**
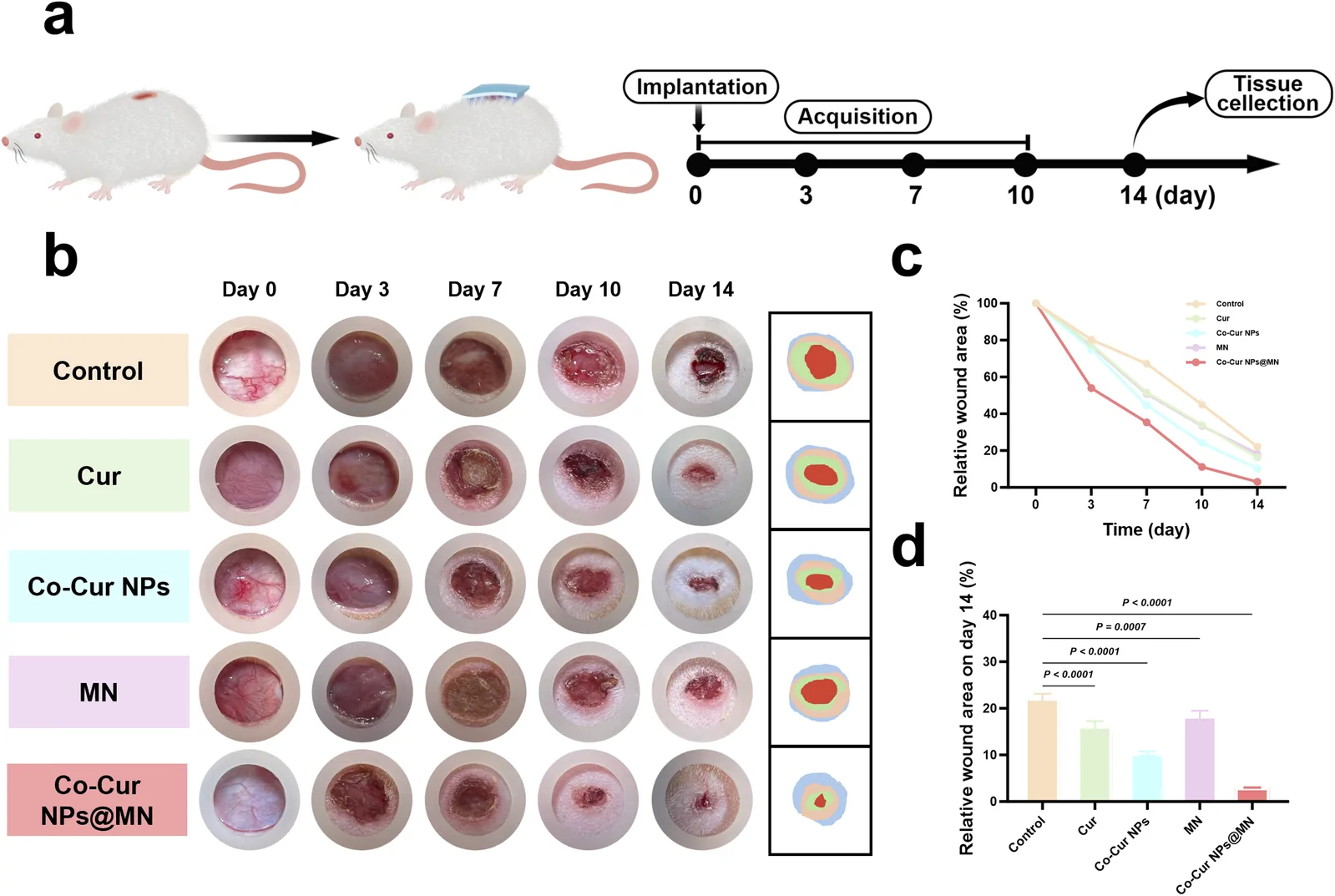
Effects of Co-Cur NPs@MN on diabetic wound closure. (a) Schematic illustration of the diabetic wound model, treatment, imaging schedule, and tissue collection. (b) Representative wound images and wound-boundary tracings for the control, Cur, Co-Cur NPs, MN, and Co-Cur NPs@MN groups on days 0, 3, 7, 10, and 14. (c) Relative wound area trajectories during the 14 day observation period. (d) Relative wound area on day 14. Measurements from two wounds in each animal were averaged to obtain one animal-level value.

Diabetic wounds, due to persistent hyperglycemia, oxidative stress, and chronic inflammation, typically exhibit severely delayed healing processes. To validate the *in vivo* therapeutic efficacy of Co-Cur NPs@MN, a full-thickness skin wound model was established in diabetic rats. As shown in Fig. 5a, circular full-thickness skin defects of 10 mm diameter were created on rat dorsal skin, and the animals were randomly assigned to the control, Cur, Co-Cur NPs, MN, and Co-Cur NPs@MN groups. Wound healing was documented photographically on days 0, 3, 7, 10, and 14, and wound areas were measured using ImageJ software. Fig. 5b presents representative images and wound-contour diagrams at the indicated time points. Results indicated that wound areas in all five groups progressively decreased over time, but with distinct healing rates. The control group exhibited the slowest wound healing, whereas the Cur and MN groups showed moderate improvements. The Co-Cur NPs group demonstrated superior wound healing compared with the control group, while the Co-Cur NPs@MN group achieved the fastest wound healing, with wounds nearly completely closed by day 14. Relative wound area curves further illustrated this pattern (Fig. 5c), and quantitative analysis on day 14 showed the lowest relative wound area in the Co-Cur NPs@MN group (Fig. 5d).

These results suggest that Co-Cur NPs@MN accelerated diabetic rat wound healing through combined material and biological effects. The improvement observed in the MN group indicates that the GelMA microneedle matrix and/or microneedle-associated physical stimulation may also contribute to wound repair. In addition, the GelMA microneedle format may improve the local contact and retention of Co-Cur NPs at the wound site compared with topical nanoparticle application.^28^ Therefore, the improved wound closure in the Co-Cur NPs@MN group may be attributed to the combined contribution of Co-Cur NPs, the GelMA microneedle matrix, and local delivery.

### 3.6 Wound tissue morphological analysis

**Fig. 6:**
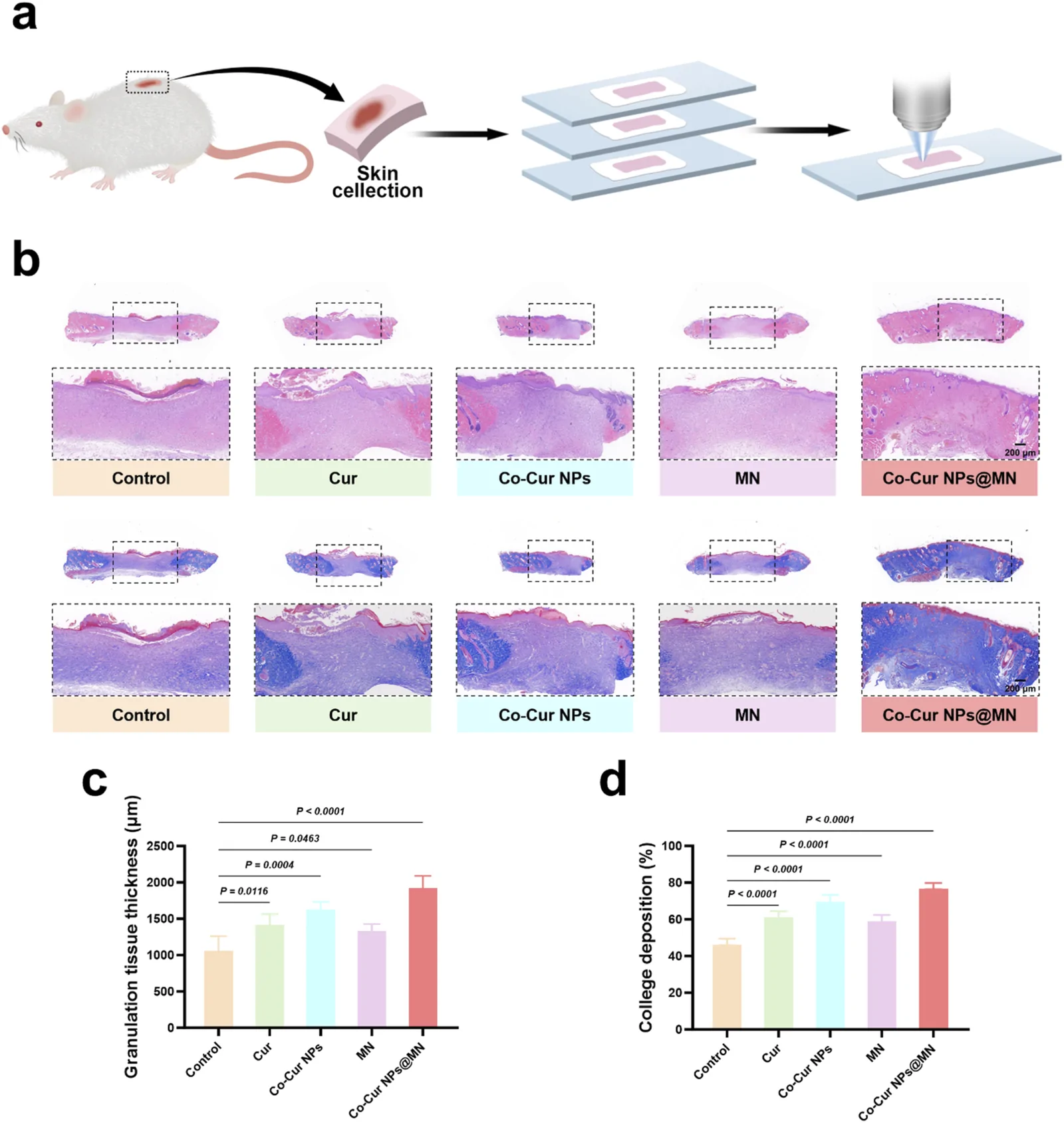
Histomorphological analysis of wound tissues. (a) Schematic illustration of wound-tissue collection, sectioning, and histological analysis. (b) Representative H&E-stained sections (upper row) and Masson's trichrome-stained sections (lower row) from the control, Cur, Co-Cur NPs, MN, and Co-Cur NPs@MN groups scale bars: 200 µm. (c) Quantification of granulation-tissue thickness. (d) Quantification of collagen deposition.

To further evaluate wound-healing quality, histological analysis of wound tissues from each group was performed. Fig. 6a illustrates the workflow for tissue harvesting, paraffin embedding, sectioning, and staining. H&E staining results (Fig. 6b, upper row) revealed incomplete epithelialization with thin, loosely structured granulation tissue in the control group. The Cur, Co-Cur NPs, and MN groups showed varying degrees of improvement, whereas the Co-Cur NPs@MN group displayed essentially complete re-epithelialization and dense granulation tissue. Quantitative analysis demonstrated that the Co-Cur NPs@MN group exhibited the greatest granulation-tissue thickness (Fig. 6c). Masson's trichrome staining (Fig. 6b, lower row) revealed sparse and disorganized collagen deposition in the control group, increased collagen deposition in the Cur, Co-Cur NPs, and MN groups, and the most abundant collagen deposition in the Co-Cur NPs@MN group. Quantitative analysis further confirmed that collagen deposition was highest in the Co-Cur NPs@MN group (Fig. 6d).

Histological analysis further supported the beneficial effect of Co-Cur NPs@MN on diabetic wound repair at the tissue level. Granulation tissue represents a critical temporary structure during wound healing, comprising neovessels, fibroblasts, and extracellular matrix, providing scaffolding for subsequent re-epithelialization and tissue remodeling.^32^ Results demonstrate that granulation tissue thickness in the Co-Cur NPs@MN group was significantly greater than in control groups, indicating that this therapeutic strategy effectively promotes granulation tissue formation. Collagen, as a major component of the extracellular matrix, plays essential roles during the proliferative and remodeling phases of wound healing.^33^ Masson staining results demonstrated not only increased collagen deposition in the Co-Cur NPs@MN group but also more organized collagen fiber arrangement. This ordered collagen arrangement may contribute to improved structural restoration of the healed tissue. The increased collagen deposition may be related to the inflammation-regulating effects of Co-Cur NPs and the resulting improvement in the wound microenvironment. Additionally, the degree of re-epithelialization in the Co-Cur NPs@MN group was notably superior to other groups, which is crucial for restoring skin barrier function. Collectively, the H&E and Masson's trichrome staining results indicate that Co-Cur NPs@MN not only promotes wound closure but also improves the histological features of healing. Recent chitosan/polypeptide and collagen/polypeptide systems have also shown wound-healing potential by providing biopolymer-based structural support and extracellular matrix-like compatibility.^39,40^ Different from these systems, Co-Cur NPs@MN integrates a redox-active cobalt-curcumin coordination nanozyme with microneedle-assisted local delivery, emphasizing oxidative-stress and inflammatory-microenvironment modulation. Therefore, these systems should be viewed as complementary wound-healing strategies with distinct material design rationales.

### 3.7 Immunofluorescence and RT-qPCR analysis of wound tissue inflammatory factors

**Fig. 7:**
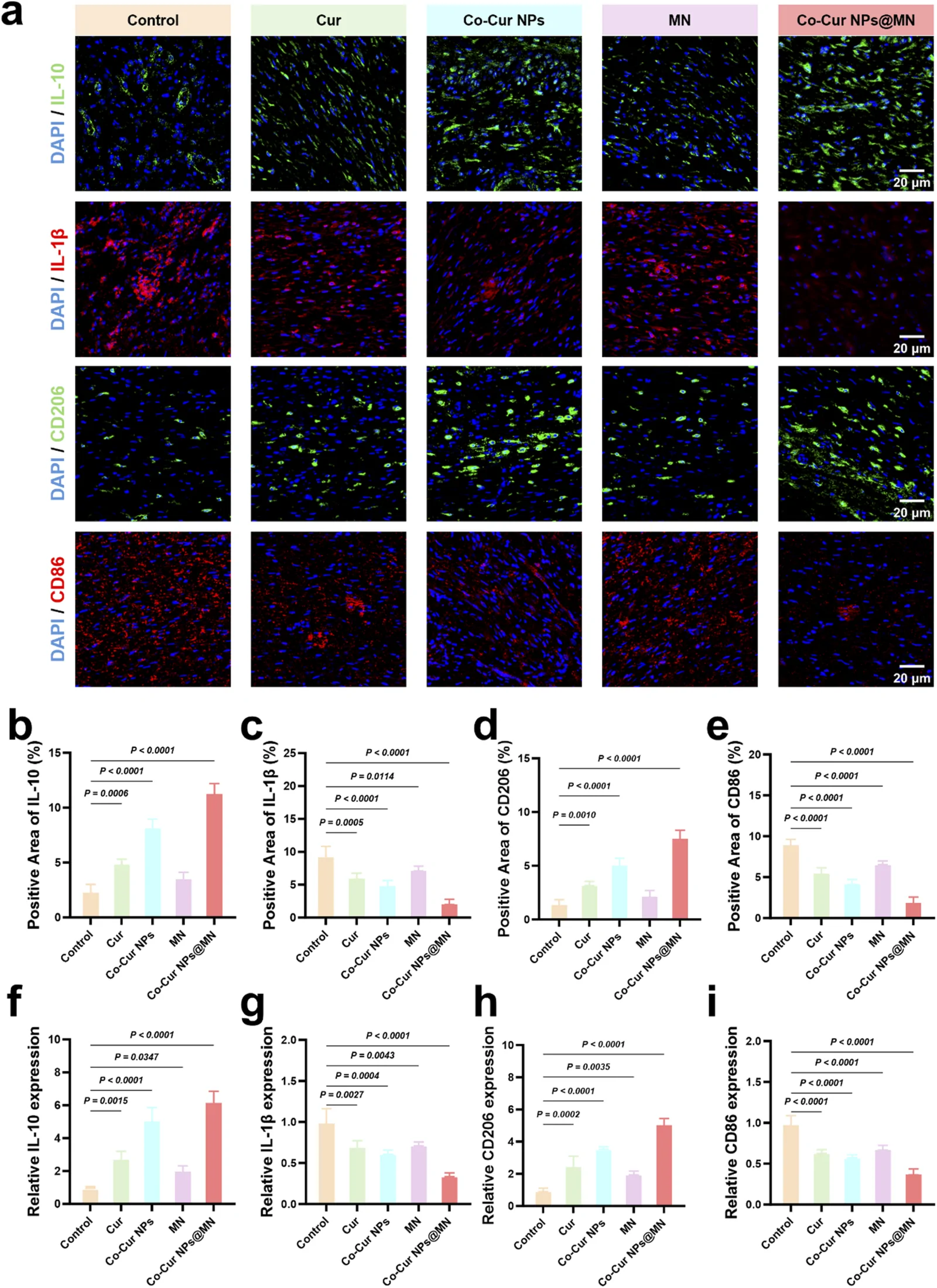
Inflammatory cytokine and macrophage-associated marker profiles in wound tissues. (a) Representative immunofluorescence images of IL-10, IL-1β, CD206, and CD86 in wound tissues from the control, Cur, Co-Cur NPs, MN, and Co-Cur NPs@MN groups. Nuclei were counterstained with DAPI scale bars: 20 µm. (b–e) Quantification of the IL-10-positive (b), IL-1β-positive (c), CD206-positive (d), and CD86-positive (e) areas. (f–i) Relative mRNA expression of IL-10 (f), IL-1β (g), CD206 (h), and CD86 (i) in wound tissues on day 14.

To validate the anti-inflammatory effects of Co-Cur NPs@MN at the *in vivo* level, immunofluorescence staining for IL-10, IL-1β, CD206, and CD86 was performed on wound tissues. As shown in Fig. 7a, the Co-Cur NPs@MN group exhibited stronger IL-10 and CD206 fluorescence signals and weaker IL-1β and CD86 signals than the control group, while the Cur, Co-Cur NPs, and MN groups showed intermediate changes. Quantitative analysis demonstrated an increased IL-10-positive area (Fig. 7b), a decreased IL-1β-positive area (Fig. 7c), an increased CD206-positive area (Fig. 7d), and a decreased CD86-positive area (Fig. 7e) following Co-Cur NPs@MN treatment. These immunofluorescence results supported the anti-inflammatory effect of Co-Cur NPs@MN at the *in vivo* level. IL-1β is a crucial pro-inflammatory cytokine that is typically highly expressed in diabetic wounds and can aggravate inflammatory responses and delay wound healing.^34^ In contrast, IL-10 is an important anti-inflammatory cytokine involved in inflammation resolution and tissue repair.^35,36^ The reduced IL-1β and CD86 signals together with the increased IL-10 and CD206 signals suggest that Co-Cur NPs@MN shifted the inflammatory wound microenvironment toward a more reparative profile. To further validate these changes at the transcriptional level, RT-qPCR was performed using wound tissues collected from each group. Co-Cur NPs@MN treatment increased IL-10 mRNA expression (Fig. 7f) and decreased IL-1β expression (Fig. 7g). Similarly, CD206 expression was increased (Fig. 7h), whereas CD86 expression was decreased (Fig. 7i). These results were consistent with the immunofluorescence findings and further supported the regulation of the inflammatory wound microenvironment by Co-Cur NPs@MN.

### 3.8 *In Vivo* biosafety evaluation of Co-Cur NPs@MN

**Fig. 8:**
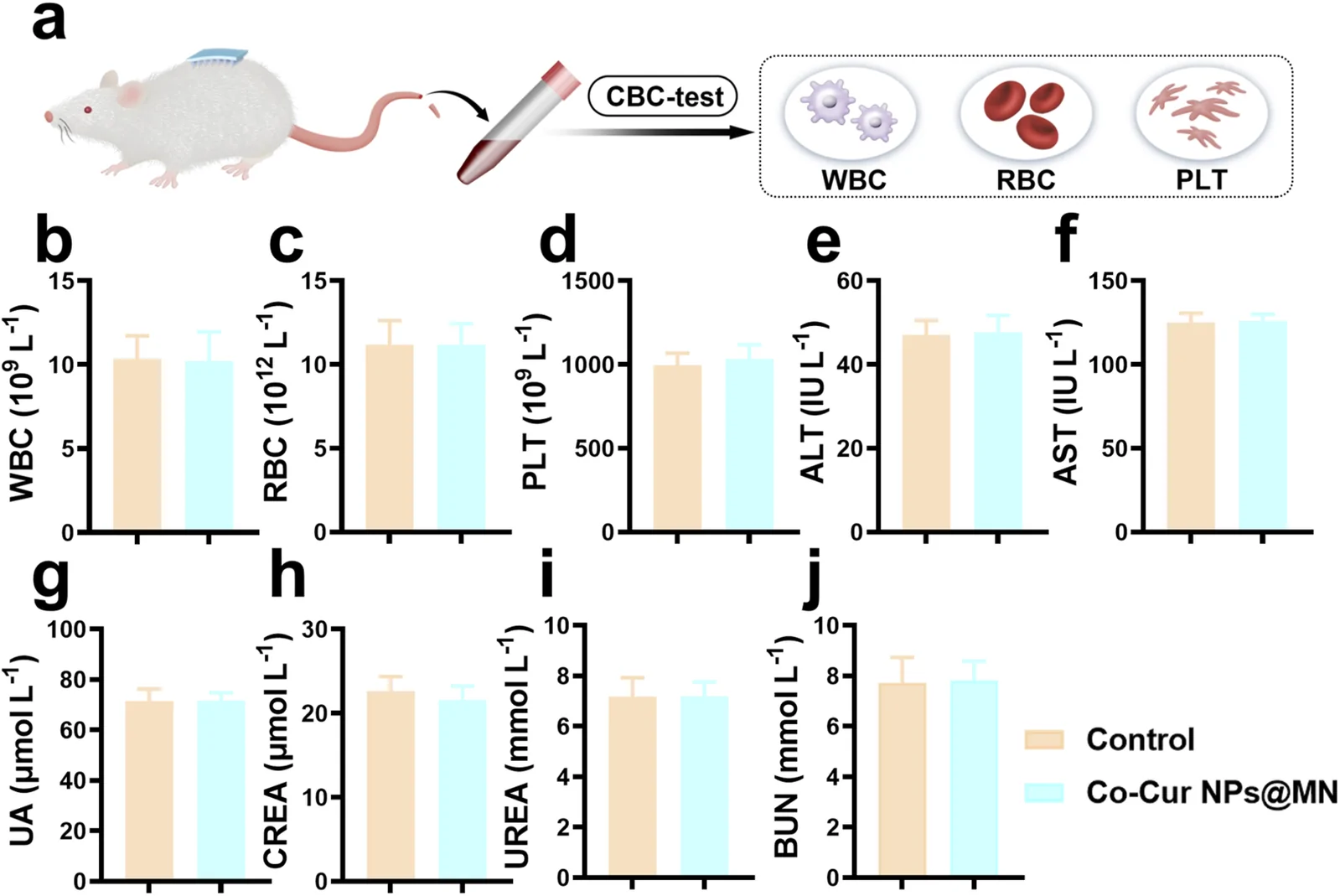
*In vivo* biosafety evaluation of Co-Cur NPs@MN. (a) Schematic illustration of blood sample collection and hematological and biochemical analysis in rats. (b–d) Hematological parameters: WBC (b), RBC (c), PLT (d). (e and f) Liver function indicators: ALT (e), AST (f). (g–j) Renal function indicators: UA (g), CREA (h), UREA (i), BUN (j).

Biosafety represents a critical consideration for nanomedicine clinical translation. To assess the systemic toxicity of Co-Cur NPs@MN, CBC and blood biochemistry analyses were performed on post-treatment rats (Fig. 8a). CBC results (Fig. 8b–d) revealed no significant differences in WBC, RBC, and PLT between the control and Co-Cur NPs@MN groups, with all parameters within normal ranges, indicating that the material does not induce hematological toxicity or immune abnormalities. Liver function indices (Fig. 8e–f) showed comparable levels of ALT and AST between groups, both within normal ranges, indicating no hepatic injury. Renal function indices (Fig. 8g–j) revealed no significant differences in UA, CREA, UREA, and BUN levels between groups, all within normal ranges, indicating no renal toxicity. The favorable biosafety profile of Co-Cur NPs@MN can be attributed to two factors. First, curcumin, as a naturally-derived plant compound, possesses high safety.^9^ Second, cobalt, as an essential trace element in the human body, does not produce toxicity at appropriate doses.^15^ Collectively, these results suggest preliminary *in vivo* biosafety of Co-Cur NPs@MN under the current experimental conditions. Because the biological effects of cobalt-containing materials depend on dose, chemical form, and release behavior, more comprehensive biosafety evaluations, including cobalt release, major organ histology, local irritation, body-weight monitoring, and longer-term observation, are still needed in future studies.

## 4. Conclusion

This study successfully constructed a Co-Cur NPs@MN delivery system for diabetic wound treatment. Co-Cur NPs prepared *via* coordination reaction exhibited uniform nano-scale dimensions, favorable colloidal stability, and excellent free radical scavenging capacity. *In vitro* experiments showed that Co-Cur NPs modulated macrophage-associated inflammatory cytokine expression, decreasing pro-inflammatory factors while increasing anti-inflammatory factors. GelMA microneedles served as a local delivery platform for Co-Cur NPs. *In vivo* experiments demonstrated that Co-Cur NPs@MN accelerated diabetic rat wound healing, promoted granulation tissue formation and collagen deposition, and alleviated the inflammatory microenvironment. CBC and blood biochemistry analyses suggested preliminary *in vivo* biosafety. Overall, Co-Cur NPs@MN represents a promising local therapeutic platform for diabetic wound repair through combined radical-scavenging and inflammatory-microenvironment modulation.

However, several limitations remain. Penetration depth, microneedle dissolution kinetics, and nanoparticle distribution within the needle tips were not directly quantified. Although free-curcumin and blank-microneedle controls were included, cobalt-only, curcumin-loaded microneedle, and standard active-treatment comparators were not evaluated, limiting component-specific attribution. The animal cohort was small, and the *in vivo* findings should therefore be considered preliminary.

## Institutional review board statement

All animal experiments in this study were approved by the Animal Ethics Committee of Yunnan Bestar Bio-Technology Co., Ltd (Approval Nos. BST-PZ-MICE-20250107-01) and were conducted in strict accordance with the Guidelines for the Use and Management of Laboratory Animals issued by the National Institutes of Health (NIH) of the USA and the Implementation Rules for the Management of Medical Laboratory Animals issued by the National Health and Family Planning Commission of China.

## Author contributions

Material preparation, experiment, data collection and analysis were performed by Gui-xuan You, Wen Chen and Dongbo Xie. The first draft of the manuscript was written by Gui-xuan You. Dongbo Xie, Fashen Huang prepared figures and table. Yang Shen made the critical revision of the manuscript. Xinke Gu contributed to the study conception and design. All authors read and approved the final manuscript.

## Conflicts of interest

The authors declare that they have no competing interests.

## Supplementary Material

RA-016-D6RA03654F-s001

## Data Availability

The data supporting the findings of this study are available within the article and its supplementary information (SI). Additional data are available from the corresponding author upon reasonable request. Supplementary information is available. See DOI: https://doi.org/10.1039/d6ra03654f.
