# Supplementary material for: Methacrylated gelatin microneedles loaded with cobalt-curcumin nanozymes promote diabetic wound healing by modulating oxidative stress and inflammatory microenvironment
Source: RSC Adv. 2026 Aug 27;16(42):50750–65. doi: 10.1039/d6ra03654f (PMC13519912; doi:10.1039/d6ra03654f)
Supplement: Supplementary file 1 [file RA-016-D6RA03654F-s001.pdf]

Supplementary Information (SI) for RSC Advances.
This journal is © The Royal Society of Chemistry 2026

Table1: Primers used for real-time PCR in this study.

Species	Gene	Forward	Reverse
Human	IL-1β	GGGACAGGATATGGAGCAACA	ACACGCAGGACAGGTACAGA
Human	IL-10	GGAGCAGGTGAAGAATGCCT	AGTCGCCACCCTGATGTCTC
Human	TGF-β	GCGTGAGTGGCTGTTGAGAAG	CATTGGGCTGAAAGGTGTGACAT
Human	GAPDH	GCACCGTCAAGGCTGAGAAC	TGGTGAAGACGCCAGTGGA
Rat	Cd86	CCAGGCTCTACGACTTCACA	GGTTTCGGGTATCCTTGCTT
Rat	Cd206	GGTGCGGTACACTAACTGGG	TTCCGTAGCCGGGATTTCGT
Rat	Il-1 β	TACCTATGTCTTGCCCGTGGA	ATCATCCCACGAGTCACAGAGG
Rat	Il-10	GGTTGCCAAGCCTTATCGGAATG	GCCGCATCCTGAGGGTCTTC
Rat	Gapdh	GGCACAGTCAAGGCTGAGAATG	ATGGTGGTGAAGACGCCAGTA

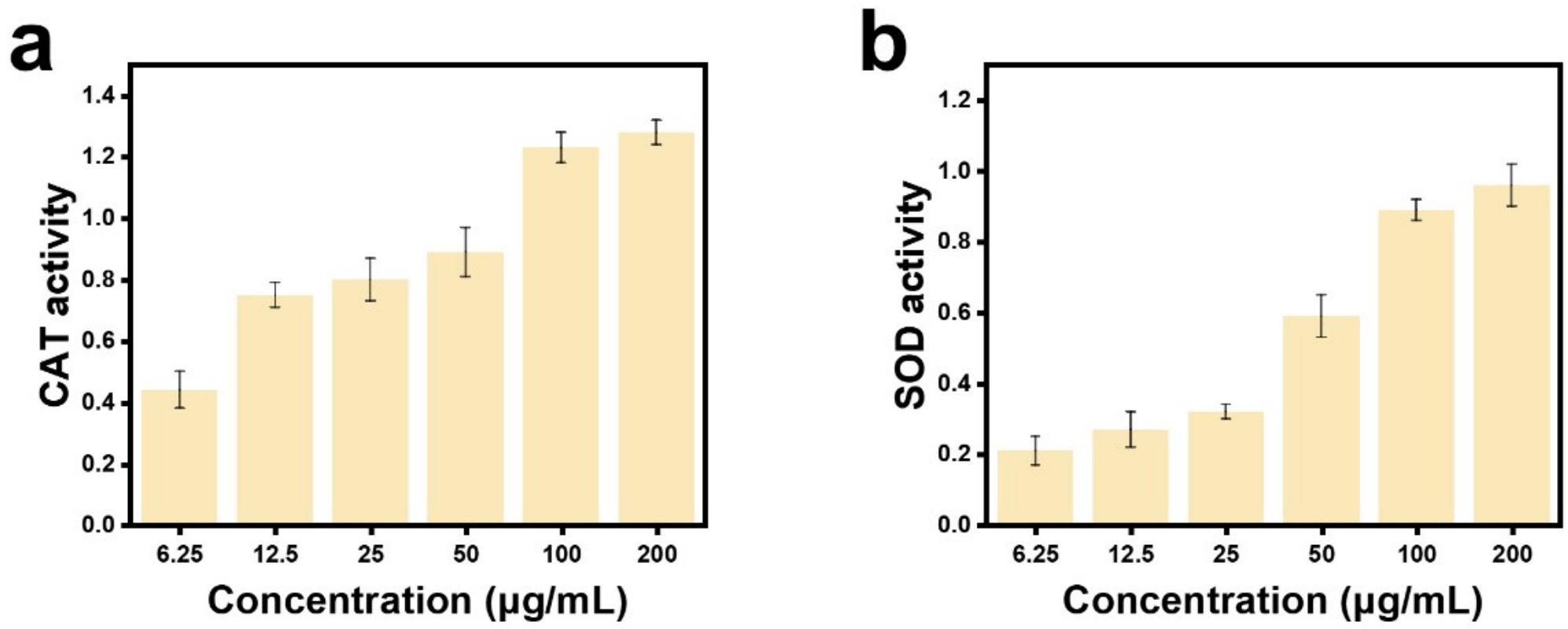

Supplementary Figure S1. Enzyme-mimetic activities of Co-Cur NPs. (a) CAT-like activity and (b) SOD-like activity of Co-Cur NPs at concentrations ranging from 6.25 to 200 μg/mL.
